# Sodium-Glucose Cotransporter 2 Inhibitors and Heart Failure: A Bedside-to-Bench Journey

**DOI:** 10.3389/fcvm.2021.810791

**Published:** 2021-12-23

**Authors:** Donato Cappetta, Antonella De Angelis, Gabriella Bellocchio, Marialucia Telesca, Eleonora Cianflone, Daniele Torella, Francesco Rossi, Konrad Urbanek, Liberato Berrino

**Affiliations:** ^1^Department of Experimental Medicine, University of Campania “Luigi Vanvitelli”, Naples, Italy; ^2^Department of Medical and Surgical Sciences, University “Magna Graecia” of Catanzaro, Catanzaro, Italy; ^3^Department of Experimental and Clinical Medicine, University “Magna Graecia” of Catanzaro, Catanzaro, Italy

**Keywords:** sodium-glucose cotransporter 2 inhibitor, heart failure, diabetes, clinical trials, sodium and calcium overload

## Abstract

Type 2 diabetes mellitus (T2DM) and heart failure (HF) are multifactorial diseases sharing common risk factors, such as obesity, hyperinsulinemia, and inflammation, with underlying mechanisms including endothelial dysfunction, inflammation, oxidative stress, and metabolic alterations. Cardiovascular benefits of sodium-glucose cotransporter 2 (SGLT2) inhibitors observed in diabetic and non-diabetic patients are also related to their cardiac-specific, SGLT-independent mechanisms, in addition to the metabolic and hemodynamic effects. In search of the possible underlying mechanisms, a research campaign has been launched proposing varied mechanisms of action that include intracellular ion homeostasis, autophagy, cell death, and inflammatory processes. Moreover, the research focus was widened toward cellular targets other than cardiomyocytes. At the moment, intracellular sodium level reduction is the most explored mechanism of direct cardiac effects of SGLT2 inhibitors that mediate the benefits in heart failure in addition to glucose excretion and diuresis. The restoration of cardiac Na^+^ levels with consequent positive effects on Ca^2+^ handling can directly translate into improved contractility and relaxation of cardiomyocytes and have antiarrhythmic effects. In this review, we summarize clinical trials, studies on human cells, and animal models, that provide a vast array of data in support of repurposing this class of antidiabetic drugs.

## Part I. At The Bedside

### Heart Failure and Diabetes: Pathophysiology and Epidemiology

Type 2 diabetes mellitus (T2DM) and heart failure (HF) are multifactorial diseases sharing common risk factors, such as obesity, hyperinsulinemia, insulin resistance, dyslipidemia, inflammation, and thrombophilia. The pathophysiologic aspects of both pathologies are closely related to the underlying mechanisms including endothelial dysfunction, inflammation, oxidative stress, and metabolic alterations (1). According to epidemiologic analyses, patients with HF have an increased risk of developing T2DM compared to the general population. Also, HF is highly prevalent in patients with diabetes (25% in chronic HF and up to 40% in acute HF), who are at a higher risk (up to 2-fold in men and 5-fold in women) of developing HF than patients without diabetes. Rather than an adverse effect of hyperglycemia on the heart, this relationship seems to be attributable more to hyperinsulinemia (2–4). Therefore, the cardiovascular (CV) implications and the effects of antidiabetic therapy on CV risk factors have been a key aspect for clinicians in the last decades, with the primary aim to prevent death and morbidity due to CV and microvascular diseases.

The first evidence of a negative CV event of a glucose-lowering agent occurred with rosiglitazone (5, 6). A concern about an increased risk of HF in people with T2DM led to the drug withdrawal from the market. Since then, several anti-diabetic drugs have been tested in CV outcome trials designed primarily to evaluate their CV safety. CV outcome trials were performed with several drug classes, such as gliptins (saxagliptin, alogliptin, sitagliptin, and linagliptin), glucagon-like peptide 1 receptor agonists (lixisenatide, liraglutide, semaglutide, exenatide, albiglutide, and dulaglutide), and sodium-glucose cotransporter 2 (SGLT2) inhibitors, the gliflozins (empagliflozin, dapagliflozin, canagliflozin, ertugliflozin, and sotagliflozin). Surprisingly, rather than simply providing a safety profile on CV risk, the results of SGLT2 inhibitors suggested significant prevention of major adverse CV events (MACE) defined as a composite of CV death, non-fatal stroke, and non-fatal myocardial infarction (7).

Inhibition of glucose transporter SGLT2 by gliflozins is a treatment strategy for the management of T2DM, with a novel mechanism of action independent of insulin. SGLT2 is a transporter molecule located in the renal proximal tubule and has a high transport capacity to reabsorb glucose together with sodium. SGLT2 inhibition promotes glycosuria, natriuresis, and diuresis, resulting in weight loss and lower blood pressure (8, 9). Gliflozins share the same mechanism of action and have a similar antihyperglycemic effect. There are differences in their selectivity for SGLT2 vs. other SGLT isoforms, although it is not known whether these differences have clinical implications (7).

In the first part of this review, we will discuss the rise of this drug class to the prominent position in the European Society of Cardiology (ESC) guidelines for HF treatment taking into account, the main clinical trials assessing the potential of SGLT2 inhibitors in patients with high CV risk (Table 1). In the second part, we will examine preclinical studies conducted to explore molecular and cellular mechanisms underlying the effects of these drugs on the CV system.

**Table 1:** Summary of clinical trials assessing the efficacy of SGLT2 inhibitors on cardiovascular and renal outcomes.

**Clinical study**	**Study design**	**Patient's disease**	**Cardiovascular outcome**	**Renal outcome**
EMPA-REG OUTCOME	Empagliflozin	Diabetes	Lower HHF and CV and non-CV deaths; non-inferiority to myocardial infarction or stroke	Reduced acute renal failure rate, non-inferiority to renal function
CANVAS program	Canagliflozin	Diabetes	Reduced rate of CV death, myocardial infarction or stroke	Lower rate of progression of albuminuria and higher rate of albuminuria regression
DECLARE-TIMI 58	Dapagliflozin	Diabetes	Reduced rate of CV death or HHF; non-inferiority to MACE	No difference in eGFR, new end-stage renal disease or death from renal causes
VERTIS CV	Ertugtliflozin	Diabetes	Non-inferiority to rate of CV death, myocardial infarction or stroke	No difference death from renal causes, renal replacement therapy, or doubling of the serum creatinine level
SCORED	Sotagliflozin	Diabetes and CKD	Lower risk of CV death, HHF and urgent visits for HF	No difference in renal function, chronic dialysis or renal transplant
CREDENCE	Canagliflozin	Diabetes and CKD	Lower risk of cardiovascular death, myocardial infarction, or stroke	Reduced risk of end-stage kidney disease, doubling of the serum creatinine level or death from renal causes
DAPA-CKD	Dapagliflozin	CKD	Reduced HHF or CV death	Lower risk of eGFR decline, end-stage kidney disease or death from renal causes
DAPA-HF	Dapagliflozin	HFrEF	Lower risk of worsening HF, CV death or HHF	No difference in the incidence of eGFR decline, end-stage renal disease or renal death
EMPEROR-Reduced	Empagliflozin	HFrEF	Lower risk of CV death or HHF	Slower decline in the eGFR
SOLOIST-WHF	Sotagliflozin	Diabetes and HF	Lower incidence of CV death, HHF or urgent visits for HF	No change in eGFR
EMPEROR-Preserved	Empagliflozin	HFpEF	Reduced risk of CV death or HHF	Slower rate of decline in the eGFR

*CKD, chronic kidney disease; CV, cardiovascular; eGFR, estimated glomerular filtration rate; HF, heart failure; HFpEF, heart failure with preserved ejection fraction; HFrEF, heart failure with reduced ejection fraction; HHF, hospitalization for heart failure; MACE, major adverse cardiovascular event*.

### Cardiovascular Outcome Trials

Five large CV outcome trials were carried out to assess the effects of SGLT2 inhibitors. These studies revealed robust cardioprotection in patients with T2DM at high risk for HF. In a large population of patients with T2DM, they showed, to a different extent, lower MACE and a reduced risk for HF hospitalization, compared to placebo (10–14).

#### Empagliflozin Cardiovascular Outcome Event Trial in Patients With T2DM

The EMPA-REG OUTCOME study was a randomized, double-blind, placebo-controlled trial to assess the effect of empagliflozin on mortality and CV morbidity in 7,020 subjects with T2DM at high risk for CV events. The patients were randomly assigned to once-daily empagliflozin (10 or 25 mg), in addition to standard care, or to placebo. The median follow-up period was of 3.1 years. The primary outcome was a composite of death from CV causes, non-fatal myocardial infarction, or non-fatal stroke. The secondary key outcome was a composite of the primary outcome plus hospitalization for unstable angina. Compared to the placebo group, empagliflozin showed a significantly lower rate of death from CV causes (38% for relative risk reduction), hospitalization for HF (35% for relative risk reduction), and death from any cause (32% for relative risk reduction). No significant differences in the rates of myocardial infarction or stroke were observed. Despite concerns about the renal safety of SGLT2 inhibitors, renal function was maintained with empagliflozin, and results regarding the new onset or worsening of nephropathy demonstrated a significant reduction (−39%) in the empagliflozin-treated group as compared to placebo. These findings proved the superiority of empagliflozin over standard care, with solid effectiveness in lowering mortality (10).

#### The Canagliflozin Cardiovascular Assessment Study Program

The Canagliflozin Cardiovascular Assessment Study (CANVAS) program integrated data from two randomized, placebo-controlled trials (CANVAS and CANVAS-R) involving 10,142 participants with T2DM and elevated CV risk. Participants in CANVAS were randomly assigned to receive daily canagliflozin (100 or 300 mg) or a matching placebo, whereas participants in CANVAS-R were assigned to receive canagliflozin (initial dose of 100 mg daily with an optional increase to 300 mg) or a matching placebo. The mean follow-up period was of 3.6 years. The primary outcome was a composite of death from CV causes, non-fatal myocardial infarction, or non-fatal stroke. Canagliflozin demonstrated superiority over the placebo concerning the primary outcome (26.9 vs. 31.5 participants per 1,000 patient-years). Canagliflozin was also associated with the amelioration of renal outcomes, with a lower rate of progression of albuminuria as well as a higher rate of albuminuria regression. All serious adverse events were lower in the canagliflozin group, although the treatment was associated with a significant increase in amputations. In summary, the CANVAS program showed a beneficial effect of canagliflozin in reducing MACE among patients with T2DM who had an increased risk of CV disease (11).

#### The Multicenter Trial to Evaluate the Effect of Dapagliflozin on the Incidence of Cardiovascular Events

The DECLARE-TIMI 58 trial was a randomized, double-blind, placebo-controlled study of dapagliflozin (10 mg daily) or a matching placebo in 17,160 patients with T2DM and established atherosclerotic CV disease or with multiple risk factors. The median follow-up was of 4.2 years. The protocol included two primary outcomes, MACE, and a composite of CV death or hospitalization for HF. Secondary outcomes were a renal composite [decrease in the estimated glomerular filtration rate (eGFR), new end-stage renal disease, or death from renal causes] and death from any cause. Dapagliflozin did not result in a lower rate of MACE than placebo (8.8 and 9.4%, respectively). However, it reduced the rate of CV death or hospitalization for HF outcome (4.9 vs. 5.8%), due to a marked impact on hospitalization for HF with no between-group differences in the CV death. The incidence of the renal outcome did not differ significantly between the dapagliflozin group (4.3%) and the placebo (5.6%). In summary, in patients with T2DM, dapagliflozin did not influence the rate of MACE or CV death with respect to placebo but resulted in a lower rate of hospitalization for HF (12).

#### Cardiovascular Outcomes Following Ertugliflozin Treatment in T2DM Participants With Vascular Disease

The VERTIS CV was a randomized, placebo-controlled, double-blind study that enrolled 8,246 patients with T2DM and high CV risk to receive ertugliflozin (5 or 15 mg) or a placebo, in addition to standard therapy. The median follow-up was of 3.5 years. The primary outcome was a composite of death from CV causes, non-fatal myocardial infarction, or non-fatal stroke. The key secondary outcomes were a composite of death from CV causes or hospitalization for HF and a composite of death from renal causes, renal replacement therapy, or serum creatinine level. The primary outcome occurred in 11.9% of patients in both the trial groups. Moreover, no significant benefit of ertugliflozin was observed for the renal composite outcome. These findings indicated the non-inferiority of ertugliflozin to standard care with respect to MACE. However, the superiority analyses for primary and secondary outcomes did not reach a statistical significance (13).

#### Effect of Sotagliflozin on Cardiovascular and Renal Events in Patients With T2DM and Moderate Renal Impairment Who Are at CV Risk

The SCORED study was a randomized, double-blind, placebo-controlled trial comparing sotagliflozin (200 mg once daily) with placebo in 10,584 patients with T2DM, chronic kidney disease (CKD), and additional CV risk. The median follow-up was of 16 months. The primary outcome was a composite of the total number of deaths from CV causes, hospitalizations for HF, and urgent visits for HF. In patients with T2DM and CKD, with or without albuminuria, sotagliflozin resulted in a lower risk than placebo (5.6 events per 100 patient-years vs. 7.5 events per 100 patient-years, respectively) of the composite of deaths from CV causes, hospitalizations for HF, and urgent visits for HF. The rate of deaths from CV causes did not change. No difference in the composite of renal function, chronic dialysis, or renal transplant was found between the groups. Adverse effects, such as diarrhea, genital infections, volume depletion, and diabetic ketoacidosis were more common with sotagliflozin than with placebo. Additionally, kidney injury did not differ significantly between the sotagliflozin and placebo groups (14). Thus, probably longer trials are required for a better evaluation of the effects and safety of sotagliflozin in patients with diabetes and CKD.

Differences regarding the CV risk profile and the phenotype of trial patients could explain the heterogeneity in the CV event incidence and treatment efficacy observed across studies. Although the consensus points to a class effect, pharmacokinetic and pharmacodynamic differences leading to dissimilar outcomes cannot be completely ruled out. Overall, the trials suggest a clear efficacy of this class of drugs on MACE in patients with diabetes. As a result, ESC guidelines for the treatment of HF, released in mid-2021, recommend all SGLT2 inhibitor class (empagliflozin, dapagliflozin, canagliflozin, ertugliflozin, and sotagliflozin) to reduce hospitalizations for HF, major CV events, and death in patients with T2DM who are at the risk of CV events (15).

### Renal Outcome

To prevent the worsening of kidney function is a key aim as T2DM is a leading cause of end-stage renal disease (16, 17). Focusing on the renal outcomes in the CV outcome trials revealed that the treatment with SGLT2 inhibitors provided benefits on the renal function, with a significant impact on acute kidney injury (empagliflozin) or death from renal causes (canagliflozin) (10–14). While in the CV outcome trials, the renal outcome was subordinate to MACE and HF hospitalization analyses, kidney disease assessment was a priority in two dedicated trials.

#### Evaluation of the Effects of Canagliflozin on Renal and CV Outcomes in Participants With Diabetic Nephropathy

The CREDENCE study was a randomized, double-blind, placebo-controlled, multicenter clinical trial assessing the effects of canagliflozin (100 mg daily) or placebo on renal outcomes in 4,401 patients with diabetic nephropathy. The primary outcome was a composite of end-stage kidney disease, doubling of the serum creatinine level, or death from renal or CV disease. The secondary outcome was a composite of CV death or hospitalization for HF. The median follow-up was of 2.6 years. The event rate of the primary outcome was significantly lower in the canagliflozin group than in the placebo group (43.2 vs. 61.2 per 1,000 patient-years, respectively). Patients in the canagliflozin group also had a lower risk to develop adverse CV events compared to placebo. Different from the CANVAS program, the rate of amputation and fracture was comparable in the canagliflozin and placebo groups and consistent with trials of other SGLT2 inhibitors. In summary, the canagliflozin treatment determined a lower risk of kidney failure and CV events among patients with T2DM and kidney disease (18).

#### A Study to Evaluate the Effect of Dapagliflozin on Renal Outcomes and CV Mortality in Patients With CKD

The DAPA-CKD study was a randomized, double-blind, placebo-controlled, multicenter clinical trial conducted in 4,304 patients with CKD, with and without T2DM, to test the effects of dapagliflozin on renal function. The median follow-up was of 2.4 years. The primary composite outcome was the eGFR decline, the onset of end-stage kidney disease, or death from renal or CV causes. The secondary outcome was a composite of hospitalization for HF or death from CV causes. The median follow-up was of 2.4 years. The occurrence of the primary outcome was lower in the dapagliflozin group (197 participants, 9.2%) than in the placebo group (312 participants, 14.5%). The rate of secondary outcome was lower in the dapagliflozin group than in the placebo group. The effect of dapagliflozin was generally consistent between diabetic and non-diabetic subgroups. The incidence of adverse events was similar in the dapagliflozin and placebo groups. Interestingly, the renoprotective effect of SGLT2 inhibitors, previously shown in the CREDENCE trial, has been extended to the broader population of patients with CKD without T2DM (19).

A third trial (EMPA-KIDNEY), testing cardio-renal protection by empagliflozin in patients with CKD in the absence of diabetes (NCT03594110), is currently ongoing.

Overall, SGLT2 inhibitors slow down the functional decline of kidney damage and prevent the progression of kidney damage. However, these drugs increase urinary tract and genital infections and can induce euglycemic ketoacidosis in patients with diabetes. Most adverse effects occur acutely and are completely reversible after drug discontinuation (20, 21). No increase in serious renal adverse events in patients with poor renal function emerges from *post-hoc* analysis and from dedicated studies on patients with CKD. Probably, the only serious adverse event in patients receiving SGLT2 inhibitors regards the increased risk of amputation with canagliflozin. However, the signal for such an effect, with the mechanism still unclear, has been detected only in one of the CANVAS studies (11).

### European Society of Cardiology Guidelines and SGLT2 Inhibitors in Non-diabetic Patients

The 2021 ESC guidelines indicate the triad composed of an angiotensin converting enzyme (ACE) inhibitor or angiotensin receptor-neprilysin inhibitor (ARNI), a beta-blocker, and a mineralcorticoid receptor antagonist (MRA) (drugs with class I recommendation) as the cornerstone therapy for all patients with HF with reduced ejection fraction (EF) (HFrEF). The use of the angiotensin receptor blocker alone is limited in patients who do not tolerate the ACE or the ARNI. Still, a substantial gap exists for patients with HF with preserved EF (HFpEF). Despite the important role of the renin-angiotensin-aldosterone system in HF pathophysiology, all the drugs blocking this system are ineffective in HFpEF. However, there is a substantial novelty introduced by 2021 ESC guidelines regarding the management of patients with HFrEF. New recommendations authorize the use of SGLT2 inhibitors, such as dapagliflozin and empagliflozin, originally developed and approved to treat T2DM as monotherapy or in combination with other glucose-lowering agents. These drugs, in addition to other class I drugs, reduce the risk of HF hospitalization and death regardless of the presence or absence of diabetes (15).

This new therapeutic indication is based on the results of two clinical trials (22, 23). The encouraging data on MACE and HF from the first SGLT2 inhibitor trials led to the design and carrying out of new clinical studies to investigate the efficacy of SGLT2 inhibitors in diabetic and non-diabetic patients with HF.

#### Study to Evaluate the Effect of Dapagliflozin on the Incidence of Worsening HF or CV Death in Patients With Chronic HF

The DAPA-HF was a multicenter, randomized, double-blind, placebo-controlled study in 4,744 patients with HFrEF (with and without T2DM) evaluating the effect of dapagliflozin (10 mg once-daily) vs. placebo, in addition to the standard of care therapy. The median follow-up was of 1.5 years. The primary outcome was a composite of worsening HF or CV death. The secondary key outcomes were a composite of hospitalization for HF or CV death in addition to a composite of eGFR decline, end-stage renal disease, or renal death. The first worsening HF event was less frequent in the dapagliflozin group than in the placebo group (10.0 vs. 13.7%, respectively). Deaths from CV causes were also significantly lower in the dapagliflozin group than in the control group (9.6 vs. 11.5%, respectively). The incidence of the renal composite outcome did not differ between the study groups. Adverse events (volume depletion, renal dysfunction, and hypoglycemia) did not differ between the groups. The major finding was that the beneficial effects in patients receiving dapagliflozin were independent of the presence or absence of diabetes (22).

#### Empagliflozin Outcome Trial in Patients With Chronic HFrEF

The EMPEROR-Reduced was a randomized, placebo-controlled, double-blind study recruiting 3,730 patients with HFrEF and testing the efficacy of empagliflozin (10 mg/day) vs. placebo in reducing CV and renal outcomes, with or without T2DM. The median follow-up was of 1.3 years. The primary outcome was a composite of CV death or hospitalization for HF. The secondary outcome was the rate of the decline in kidney function. The primary outcome events were less frequent after empagliflozin treatment, occurring in 19.4% of the empagliflozin group, compared to 24.7% of the placebo group. The decline in the eGFR was slower in the empagliflozin group than in the placebo group (−0.55 vs. −2.28 ml per min per 1.73 m^2^ of body-surface area per year). Uncomplicated genital tract infection was reported more frequently in the empagliflozin group. The frequency of hypoglycemia and the amputation fracture did not differ between groups. The effects of empagliflozin on both CV and renal outcomes were consistent in the subgroup of patients with HF regardless of the presence or absence of diabetes (23).

As adjunctive measures indicated in the ESC guidelines, dapagliflozin, empagliflozin, and sotagliflozin are recommended in patients with diabetes and HFrEF to reduce hospitalizations for HF and CV death. Conversely, the DPP-4 inhibitor, saxagliptin, is not recommended in diabetic patients with HF, since it was observed to increase HF hospitalization; no difference over placebo for HF events was found with alogliptin, sitagliptin, and linagliptin (24). The inclusion of sotagliflozin as a treatment option in patients with T2DM and HF is a consequence of the results that emerged from a recently published clinical trial.

#### Effect of Sotagliflozin on CV Events in Patients With T2DM Postworsening HF

The SOLOIST-WHF study was a double-blind, randomized, placebo-controlled trial that tested the effect of sotagliflozin (200 mg once daily) or placebo in 1,222 patients with T2DM who were recently hospitalized for worsening HF. The median follow-up was of 9.0 months; the trial ended early because of financial concerns. The primary outcome was the total number of deaths from CV causes, hospitalizations, and urgent visits for HF. In patients with diabetes and acute decompensated HF, sotagliflozin therapy resulted in a significantly lower incidence of the primary end-point than placebo. The study also intended to assess whether the benefits of SGLT2 inhibition could be extended to HFpEF patients, did not reach any conclusion for the early discontinuation of the trial (25).

### Sodium-Glucose Cotransporter 2 Inhibitors in Patients With HFpEF

Treatment options in patients with HFpEF are limited and not as effective as in HFrEF. The results were disappointing from a large-scale trial demonstrating no strong efficacy from drugs that were able to counteract the functional decline of the heart with an EF of <40%. A recent study assessing the efficacy and safety of sacubitril/valsartan combination in patients with HFpEF (PARAGON-HF) missed its primary endpoint; however, a *post-hoc* analysis indicated a possible effect in patient subgroups who may benefit in terms of hospitalization and CV death (26). The EMPEROR-Preserved study represents the first successful trial that demonstrated treatment efficacy in HFpEF.

#### Empagliflozin Outcome Trial in Patients With Chronic HFpEF

The EMPEROR-Preserved was a randomized, double-blind, placebo-controlled trial to evaluate the effects of SGLT2 inhibitor, empagliflozin (10 mg once daily), on major HF outcomes in patients with HFpEF. The median follow-up was of 2.2 years. The primary outcome was a combined risk of CV death or hospitalization for HF. One of the secondary outcomes was the rate of decline in the eGFR during treatment. The results showed a lower occurrence of the primary composite outcome event in the empagliflozin group (13.8%) than in the placebo group (17.1%). Additionally, the rate of decline in the eGFR was slower in the empagliflozin group than in the placebo group (−1.25 vs. −2.62 ml per min per 1.73 m^2^ per year). The effect of empagliflozin on the incidence of primary outcome events was consistent between patients with or without diabetes at baseline (27).

On the other hand, the EMPERIAL-Preserved trial documented that the SGLT2 inhibitor empagliflozin did not achieve the primary endpoint of exercise ability improvement according to the 6-min walk test in patients with chronic HFpEF. This study replicates what was found in a previous trial evaluating the effect of empagliflozin on the exercise capacity in HFrEF patients (EMPERIAL-Reduced) (28).

Regarding dapagliflozin, several studies on patients with HFpEF condition have been planned. The DELIVER trial (NCT03619213) is an ongoing study aiming to recruit approximately 6,000 patients with HFpEF to be randomized to dapagliflozin or placebo in addition to the standard therapy. The primary outcome is a composite of CV death and HF events (hospitalizations for HF or urgent HF visits). The DETERMINE-Preserved (NCT03877224), a small trial (500 participants) assessing the dapagliflozin efficacy on improving the exercise capacity, has been completed and results are awaiting. Further, PRESERVED-HF, CAMEO-DAPA and STADIA-HFpEF studies (NCT03030235, NCT04730947, and NCT04475042) are small-scale randomized trials on dapagliflozin and HFpEF now recruiting patients.

## Part I: Conclusions

Clinical evidence has recognized that SGLT2 inhibitors, such as dapagliflozin and empagliflozin, substantially improve the prognosis of patients with HFrEF due to their high effectiveness on CV and renal outcomes independent of diabetes status and with mechanisms other than glucose lowering. However, these drugs have been used in clinical trials on top of beta-blockers, ACE inhibitors/ARBs, or MRAs, and the guidelines also suggest their use in combination; therefore standard CV medications still represent the gold standard therapy in clinical practice. It is conceivable that soon SGLT2 inhibitors may be considered as the first-line therapy and an auxiliary weapon in HF treatment. On the other hand, results regarding HFpEF are promising considering that, to date, ESC guidelines only recommend the screening and treatment of CV and non-CV comorbidities. Although our knowledge has significantly expanded over the past decade, many questions regarding HFpEF pathophysiology, diagnosis, and treatment remain unresolved.

## Part II. Back To The Bench

**Figure 1:**
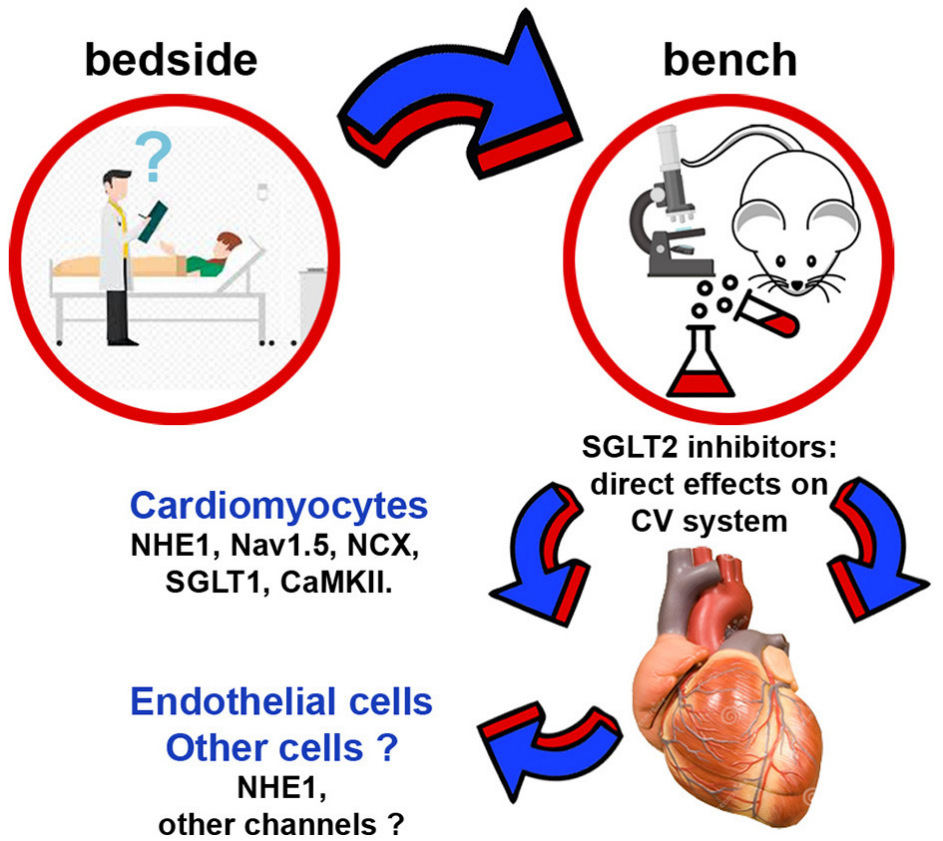
Schematic representation of non-renal cellular and molecular targets regulated by SGLT2 inhibitors. CaMKII, Ca^2+^/calmodulin-dependent protein kinase II; NCX, Na^+^/Ca^2+^ exchanger; NHE1, sodium-hydrogen exchanger 1; SGLT1, sodium-glucose cotransporter 1.

At the first glimpse, SGLT2 inhibitors appear to have relatively simple pharmacodynamics where benefits arise from the increased urinary glucose excretion and osmotic diuresis. However, these actions of SGLT2 inhibitors cannot explain their benefits on HF. The data discussed above attracted the attention and fueled the curiosity of researchers with an interest in the mechanistic aspects underlying the clinical data. This led to the identification of several molecular and cellular mechanisms by which SGLT2 inhibitors protect the patient's CV system (Figure 1).

### Beyond SGLT2: Cardiomyocyte ion Homeostasis

Abnormal cardiac Na^+^ handling is a well-recognized factor in HF progression (29). The importance of sodium-hydrogen exchange across the cell membrane is often in the research spotlight because cardiomyocyte excitation-contraction coupling and mitochondrial metabolism respond to the changes in the intracellular Na^+^ that are also linked to Ca^2+^ homeostasis *via* sarcolemmal Na^+^/Ca^2+^ exchanger (NCX) (30). Sodium-hydrogen transport is mediated by the sodium-hydrogen exchanger (NHE), an antiporter that includes several isoforms, with NHE1 being a principal isoform in the heart. NHE1 couples the extrusion of one H^+^ with the entry of one Na^+^, thus regulating intracellular pH and volume. Experimental and clinical data document increased activity of cardiac NHE1 in HF (31, 32). Activation of cardiac and vascular NHE1 may also be coupled with the maladaptive neurohormonal activation (sympathetic nervous, renin-angiotensin-aldosterone, and natriuretic peptide systems) during the pathophysiological course toward HF (33–35). The resulting increase in the intracellular sodium in cardiomyocytes enhances intracellular calcium, leading to an increase in cardiomyocyte injury and dysfunction. In addition to the elevation in NHE1 activity, cardiomyocyte intracellular sodium overload, as a result of skewed inflow-outflow balance, may also result from increased activity of channels that mediate a sustained Na^+^ current termed, “late INa” (36). The increase in late INa is a recognized source of sodium overload in HF and represents a valid molecular target in different models of HF (37–39).

While there is evidence that SGLT2 and renal isoform of NHE (NHE3) are functionally connected (40), it seems that a similar phenomenon would not take place in the heart as human cardiac cells do not express SGLT2 (35). This lack of expression in the heart together with a glucose-independent effect of SGLT2 inhibitors indicates the existence of additional molecular targets. Indeed, SGLT2 inhibitors show a strict interaction with human and murine cardiomyocytes through direct inhibition of the myocardial isoform of NHE (41, 42). *In silico* studies show that SGLT2 inhibitors bind to the Na^+^-binding pocket of NHE1 demonstrating that NHE1 is a receptor molecule for this drug class (42). Evidence that effects of reducing intracellular calcium and sodium concentrations were mediated by blocking the NHE-dependent ion flux, was supported by the use of cariporide, an established inhibitor of the NHE that annulated the results obtained from SGLT2 inhibitor (43). Notably, favorable effects of SGLT2 on cardiac function and remodeling were clearly present also in HF models in the absence of diabetes (44, 45). Moreover, late INa augmentation in HF depends on Na^+^ channel phosphorylation by Ca^2+^/calmodulin-dependent protein kinase II (CaMKII) whose activity was reduced upon SGLT2 inhibitor treatment (46). While it is likely that SGLT2 inhibitors interact with CaMKII activity in an indirect manner, a direct effect on late INa is also reasonable. In a diabetic model, the SGLT2 inhibitor, empagliflozin inhibited cardiac late INa (47). Also, the *in silico* approach confirmed the notion that empagliflozin can reduce late INa by showing that SGLT2 inhibitors can be ligands for the Nav1.5 sodium channel that drives the late INa current (48). The potential involvement of other Nav channel isoforms awaits exploration.

The interaction of SGLT2 inhibitors with ion-handling system can be of relevance, because a reduced risk of ventricular arrhythmias and sudden cardiac deaths (signs of a modulation of ion-handling system) was detected in CV safety trials. Ventricular arrhythmias are common in HF and are one of the key causes of death in HFrEF. The possibility that SGLT2 inhibitors may also possess unexpected benefits against ventricular arrhythmias emerges from *post-hoc* analysis of the DAPA-HF trial (that included also non-diabetic subjects), showing a reduction in the outcome composed of ventricular arrhythmias, resuscitated cardiac arrest, or sudden death (49). Additionally, the analysis of over 30 trials in patients with T2DM treated with canagliflozin, dapagliflozin, empagliflozin, or ertugliflozin shows the association with significantly reduced risks of atrial arrhythmias and sudden death (50). At present, we do not know whether the antiarrhythmic effects of SGLT2 inhibitors result primarily from their effects on cardiomyocyte ion homeostasis or from the benefits of structural remodeling and favorable loading conditions.

Although SGLT2 inhibitors have a significant selectivity for SGLT2, their activity on SGLT1 cannot be completely disregarded because SGLT1 is abundant in the human heart, whereas SGLT2 is barely detectable. Indeed, SGLT1 expression is positively related to myocardial oxidative stress, and the experiments in human myocardium show that a member of SGLT2 inhibitors, canagliflozin, with a known affinity to SGLT1, suppresses redox-sensitive pro-inflammatory and pro-apoptotic signaling. Mechanistically, the effects of canagliflozin on cardiomyocytes, *via* the SGLT1 signaling, lead to increased tetrahydrobiopterin bioavailability and improved coupling of nitric oxide synthase (51).

In summary, this synthetic overview presents evidence that SGLT2 inhibitors directly reduce intracellular Na^+^ accumulation in the myocardium. In addition to glucose excretion and diuresis, the benefits of SGLT2 inhibitors in HF are likely mediated by the regulation of intracellular sodium homeostasis. In this way, the restoration of cardiac Na^+^ levels with consequent positive effects on Ca^2+^ handling can directly translate into improved contractility and relaxation of cardiomyocytes and have antiarrhythmic effects. Since the increase in intracellular Na^+^ and Ca^2+^ are considered initial hallmarks of HF, the clinical impact of the normalization of ion homeostasis can be remarkable.

Oxidative stress, which is mechanistically linked to intracellular Na^+^ accumulation, has been known for decades as a major player in HF pathophysiology. Also, SGLT2 inhibitors evoke anti-oxidative effects in the heart, and the decrease in oxidative stress was linked to the downregulation of NADPH oxidase 4 and nuclear respiratory factor signaling. Reduced oxidative stress was linked to the effects of mitochondrial membrane potential, ATP content, and mitochondrial fusion/fission balance. The levels of several classical markers of oxidative stress and inflammatory process responded to SGLT2 inhibitor treatment (52–54). Moreover, *in vivo* and *in silico* studies strongly suggested an antiapoptotic effect of SGLT2 inhibitors. Repression of AKT serine/threonine kinase 1/3 and baculoviral IAP repeat containing protein (BIRC) 2 and expression of antiapoptotic X-linked inhibitor of apoptosis and BIRC were linked to NHE activation (55). Thus, inhibiting Na^+^-influx by SGLT2 inhibitors might mitigate oxidative stress and cell death that are central players in cardiac pathogenesis. The protection from myocardial cell death following the inhibition of SGLT2 can also be envisioned *via* the restoration of the physiological mammalian target of rapamycin (mTOR) activation-inhibition cycles related to nutrient availability as the modulation of the mTOR signaling pathway, apart from energy metabolism, can be linked to the regulation of apoptosis and tissue repair (56, 57).

Further benefit from the normalization of sodium dynamics can be linked to mitochondrial physiology. In HF, high cytoplasmic Na^+^ and Ca^2+^ concentrations lead to decreased mitochondrial Ca^2+^. This pathophysiological mechanism involves the action of the mitochondrial NCX that extrudes mitochondrial calcium when cytosolic sodium increases (58). A deficit in energy production and reduced antioxidant defense, known as contributors to HF progression, can be counteracted by the elevation of the mitochondrial calcium (59, 60). There is a possibility that following the treatment with SGLT2 inhibitors, a decrease in the cellular sodium load improves mitochondrial function and reduces the excessive reactive oxygen species formation (43). Also, the enhanced mitochondrial energy output, *via* the induction of oxidative phosphorylation and fatty acid metabolism genes (61), may contribute to the improved myocardial function.

### Cardiac Effects of SGLT2 Inhibitors: Beyond Cardiomyocytes

Extending the effects of drugs acting on the heart beyond cardiomyocytes broadens a pathophysiological perspective of a drug-disease interaction (62, 63). This can be of particular importance in the view of the concept that HF pathogenesis combines chronic proinflammatory state and microvascular endothelial cell dysfunction. There is evidence that also SGLT2 inhibitors act on cellular targets other than cardiomyocytes. The relevance of the sodium-hydrogen exchange mechanism in vascular biology is known and the recently identified vascular effects of SGLT2 inhibitors are supposed to be mediated by NHE inhibition. The involvement of coronary endothelium in the beneficial effects of SGLT2 inhibitors was demonstrated also in non-diabetic conditions. Endothelial upregulation of NHE1 and direct effects on SGLT2 inhibitors on the activity of this exchanger in endothelial cells were reported (42, 44, 45, 64–66).

### Renal Effects of SGLT2 Inhibitors: Beyond SGLT

As addressed in all the major trials discussed above, SGLT2 inhibitors show a protective role by preserving renal function in CKD with or without T2DM. As with the HF, pure inhibition of SGLT2 can be insufficient to explain the reduced risk of renal failure observed in patients. To date, the predominant mechanistic explanation proposes the active participation of NHE3. Several studies suggest functional coordination between the activities of SGLT2 and NHE3 in the proximal tubule (67, 68). NHE3, with the expression predominantly in the renal (and gastrointestinal) epithelial cells, mediates the majority of the sodium reuptake that follows glomerular filtration (69). Noteworthy, hyperglycemia and hyperinsulinemia stimulate the activity of renal NHE3 that is responsible for glomerular dysfunction in the genesis of diabetic nephropathy and diabetic-dependent HF (70–72). SGLT2 activation increases NHE3-dependent sodium transport (73); on the other hand, inhibition of SGLT2 results in the reduction of NHE3 activity, contributing to the natriuretic effects of SGLT2 inhibitors (74). This strict crosstalk implicates compensatory responses and metabolic adaptations observed after the administration of SGLT2 inhibitors that may result from reduced NHE3 activity (75). There is also a possibility, not yet clarified, that this class of drugs can directly bind to and inhibit NHE3, in the same way as with the cardiac isoform, NHE1.

## Conclusions: Part II

The story of SGLT2 inhibitors and unexpected HF benefits underlines the importance of continuous reverse translation research. Thanks to the scientific curiosity and laboratory effort, we learned that initially thought to act through extracardiac mechanisms, the SGLT2 inhibitors directly target the cardiac cells. Moreover, the projected primary molecular target turned out not to be the only receptor for these drugs. The intense experience with SGLT2 inhibitors shows that the genuine interest in the bedside-to-bench approach, although not common, can yield insights of the unprecedented value.

## Author Contributions

DC and KU: conceptualization and writing. DC, GB, MT, and KU: literature collection and visualization. AD, EC, DT, FR, and LB: review and editing. All authors contributed to the article and approved the submitted version.

## Funding

This review was supported by the Italian Ministry of Education, the University and Research grants [PRIN-2017XZMBYX (AD), PRIN-2017NKB2N4 (LB)].

## Conflict of Interest

The authors declare that the research was conducted in the absence of any commercial or financial relationships that could be construed as a potential conflict of interest.

## Publisher's Note

All claims expressed in this article are solely those of the authors and do not necessarily represent those of their affiliated organizations, or those of the publisher, the editors and the reviewers. Any product that may be evaluated in this article, or claim that may be made by its manufacturer, is not guaranteed or endorsed by the publisher.
